# Improvement of Intestinal Absorption of Forsythoside A and Chlorogenic Acid by Different Carboxymethyl Chitosan and Chito-oligosaccharide, Application to *Flos Lonicerae* - *Fructus Forsythiae* Herb Couple Preparations

**DOI:** 10.1371/journal.pone.0063348

**Published:** 2013-05-13

**Authors:** Wei Zhou, Haidan Wang, Xuanxuan Zhu, Jinjun Shan, Ailing Yin, Baochang Cai, Liuqing Di

**Affiliations:** 1 College of Pharmacy, Nan’jing University of Chinese Medicine, Nan’jing, People’s Republic of China; 2 Jiang’su Engineering Research Center for Efficient Delivery System of TCM, Nan’jing, People’s Republic of China; 3 Nan’jing Engineering Research Center for Industrialization of Chinese Medicine Pellets, Nan’jing, People’s Republic of China; 4 Department of Pharmacology, Affiliated Hospital of Nan’jing University of Chinese Medicine, Nan’jing, People’s Republic of China; 5 First Medicine College, Nan’jing University of Chinese Medicine, Nan’jing, People’s Republic of China; Concordia University Wisconsin, United States of America

## Abstract

The current study aims to investigate the effect of chitosan derivatives on the intestinal absorption and bioavailabilities of forsythoside A (FTA) and Chlorogenic acid (CHA), the major active components in *Flos Lonicerae* - *Fructus Forsythiae* herb couple. Biopharmaceutics and pharmacokinetics properties of the two compounds have been characterized *in vitro*, *in situ* as well as in rats. Based on the identified biopharmaceutics characteristics of the two compounds, the effect of chitosan derivatives as an absorption enhancer on the intestinal absorption and pharmacokinetics of FTA and CHA in pure compound form as well as extract form were investigated *in vitr*o, *in situ* and *in vivo.* Both FTA and CHA demonstrated very limited intestinal permeabilities, leading to oral bioavailabilities being only 0.50% and 0.13% in rats, respectively. Results from both *in vitro*, *in situ* as well as *in vivo* studies consistently indicated that Chito-oligosaccharide (COS) at dosage of 25 mg/kg could enhance intestinal permeabilities significantly as well as the *in vivo* bioavailabilities of both FTA and CHA than CMCs in *Flos Lonicerae* - *Fructus Forsythiae* herb couple preparations, and was safe for gastrointestine from morphological observation. Besides, treatment with *Flos Lonicerae* - *Fructus Forsythiae* herb couple preparations with COS at the dosage of 25 mg/kg prevented MDCK damage after influenza virus propagation, which was significantly better than control. The current findings not only identified the usefulness of COS for the improved delivery of *Flos Lonicerae* - *Fructus Forsythiae* preparations but also demonstrated the importance of biopharmaceutical characterization in the dosage form development of traditional Chinese medicine.

## Introduction

**Figure 1:**
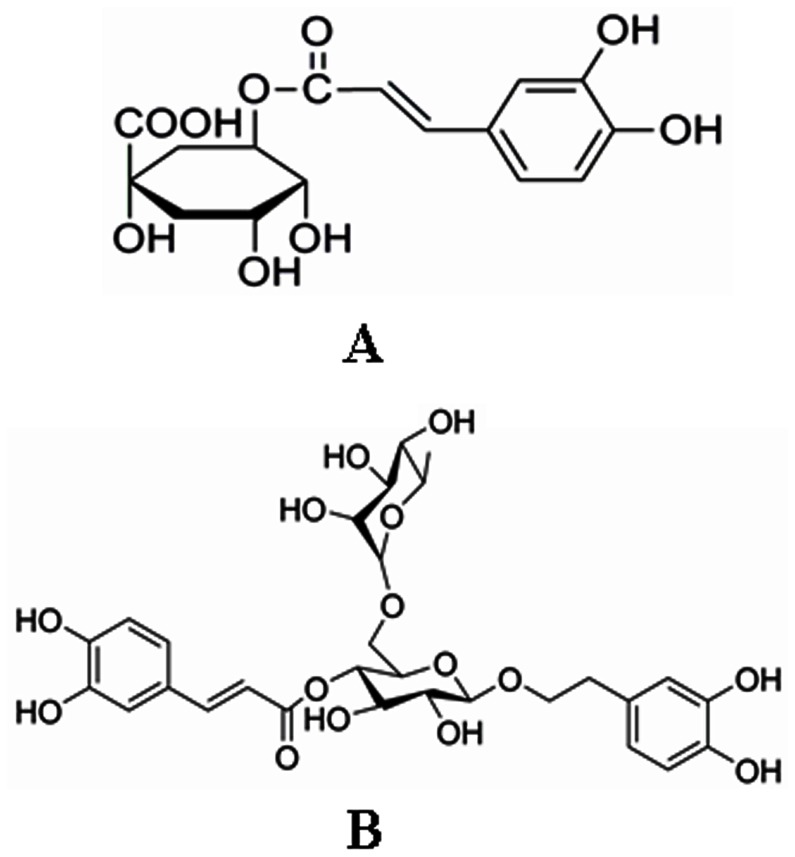
Chemical structure of FTA and CHA (A: CHA; B: FTA).

Herbs used together in couples are the basic composition units of Chinese herbal formulas and have special clinical significance in Traditional Chinese Medicine (TCM). The herb couples (mixture of two herbs) are much simpler than complicated formulations in composition but retain the basic therapeutic features. *Flos Lonicerae* possesses wide pharmacological actions, such as antibacterial, anti-inflammatory, antiviral, antiendotoxin, blood fat reducing, antipyretic, *etc* [1]. And *Fructus Forsythiae* has the effects of antibacterial, antiviral, antioxidant, anti-inflammatory, anti-obesity and antiemetic, *etc* [2]. The two herbs are the basic components of Chinese herbal preparations such as Shuang-Huang-Lian tablet, Yin-Qiao-Jie-Du tablet, Fufang Jin-Huang-Lian Granule, Qin-Re-Jie-Du oral liquid and Fufang Qin-Lan oral liquid *etc*., shown in Table 1, which are extensively used in clinical practice. Findings from us and others consistently demonstrated that the hydrophilic components e.g. forsythoside A (FTA) and chlorogenic acid (CHA) (Fig. 1) were the main components in the commercial *Flos Lonicerae* - *Fructus Forsythiae* herb couple preparations [1], [2]. Pharmacological studies demonstrated that FTA possessed strong antioxidant, antibacterial and antiviral activities, and also exhibited a slow relaxation effect against norepinephrine-induced contraction of rat aorta [3], [4]. Moreover, it was reported that FTA could significantly protect DNA damage caused by hydroxyl redicals and inhibit protein kinase C (PKCα) with an IC_50_ value of 1.9 µM [5]. The main pharmacological activities of CHA included: stronger bacteriostasis activity against gram-negative bacteria than against gram-positive bacteria, significant antiviral activity to respiratory syncytia virus, coxsackie B3 virus, adeno-associated 7 viruses, adeno-associated 3 viruses and coxsackie B5 virus respectively [6]. Besides, it was reported that CHA had antioxidant and anti-inflammatory activity [7].

**Table 1:** The traditional and clinical uses of *Flos Lonicerae* - *Fructus Forsythiae* herb couple preparations.

Preparation name	Main compositions	Traditional and clinical uses	References
Shuang-Huang-Lian tablet	*Flos Lonicerae, Fructus Forsythiae, Radix Scutellariae*	Curing upper respiratory tractinfection, pneumonia, fever,cough and pharyngalgia.	Chinese Pharmacopoeia
Yin-Qiao-Jie-Du tablet	*Flos Lonicerae, Fructus Forsythiae, Herba Menthae* *Haplocalycis, Herba Schizonepetae, Fructus Arctium,* *Radix Platycodi, Folium Lophatheri, Radix Glycyrrhizae*	Curing the swell of throat, constipation,conjunctival congestion, *etc*	Chinese Pharmacopoeia
Fufang Jin-Huang-LianGranule	*Flos Lonicerae, Fructus Forsythiae, Radix Scutellariae,* *Radix Isatidis, Taraxacum*	Curing fever, cough, pharyngalgia, *etc*	Chinese Pharmacopoeia
Qin-Re-Jie-Du oral liquid	*Flos Lonicerae, Fructus Forsythiae, Gypsum Fibrosum,* *Radix Scrophulariae, Radix Rehmanniae, Fructus* *Gardeniae, Radix Scutellariae, Radix Gentianae, Radix* *Isatis, Rhizoma Anemarrhenae, Radix Ophiopgonis*	Clearing away the heat-evil andexpelling superficial evils	Chinese Pharmacopoeia
Fufang Qin-Lan oral liquid	*Flos Lonicerae, Fructus Forsythiae, Radix Scutellariae,* *Radix Isatidis*	Curing fever, cough, pharyngalgia, *etc*	Http://www.sfda.gov.cn

Due to the promising pharmacological effects of FTA and CHA, more researches started to investigate their pharmacokinetics properties. Pharmacokinetics of CHA in pure form in rats has been investigated by us showing that *T*_1/2z_ was 63 min, *C*_max_ was 200 ng/mL, *T*_max_ was 15 min, *AUC*_0→∞_ of 9664.2 ng⋅min/mL and the absolute bioavailability was about 0.13% after oral administration of 60 mg/kg CHA (unpublished). And the pharmacokinetics of FTA in pure form in rats has been found by Wang et al. (2010) [8] showing that *T*_1*/2λz*_ was 74.7 min, *C*_max_ was 122.2 ng/mL, *T*_max_ was 20 min, *AUC*_0→∞_ of 14600 ng⋅min/mL and the absolute bioavailability was about 0.5% after oral (100 mg/kg) administration. Besides, it was reported by Ye et al. (2010) [9] that the pharmacokinetic parameters of CHA and FTA were described by a non-compartmental model with a *T*_1*/2λz*_ being 218 min and 403 min, *C*_max_ being 33.8 ng/mL and 35 ng/mL, *T*_max_ being 45 min and 45 min, *AUC*_0→∞_ being 6970 ng⋅min/mL and 10200 ng⋅min/mL, respectively after oral administration of 1000mg/kg Shuang-Huang-Lian freeze-dried powders of injectable grade (equivalent to 10 mg/kg of CHA and 17 mg/kg of FTA ) in rats, and their absolute bioavailabilities orally were about 0.69% and 0.72%, respectively. In summary, the previous studies consistently suggested a poor intestinal absorption of FTA and CHA, which influenced the efficacy of *Flos Lonicerae* - *Fructus Forsythiae* herb couple preparations in clinical practice seriously.

According to our previous study, the intestinal absorption mechanism of CHA and FTA was passive diffusion, and involved paracellular route transport mainly governed by the tight junctions (TJs) [10], [11], and the modulation of the TJs by absorption enhancers would enhance the paracellular drug transport [12].

It was reported that absorption enhancers including surfactants, bile salts and chelating agents *etc.* were one of the most promising methods to improve the bioavailability of poorly absorbable drugs orally [13]. Recently, it was demonstrated that nitric oxide (NO) donors and polyamines were also effective for improving the intestinal absorption of poorly absorbable drugs [14]. However, some absorption enhancers could cause damage and irritate the intestinal mucosal membranes. This was a limiting factor for their clinical use. Indeed, there existed almost linear relationship between the effectiveness of various absorption enhancers and their membrane toxicity reported by Yamamoto et al. (1996) [15]. Therefore, the absorption enhancers based on tight junctions with high effectiveness and low mucosal toxicity need to be investigated.

Chitosan, a natural polymer obtained by alkaline deacetylation of chitin, is non-toxic, biocompatible, and biodegradable. The ability of chitosan to enhance gastrointestinal (GI) drug absorption has been of special interest [16]. However, the polymer was only soluble in an acidic environment in which the pH was less or if the order of the pKa value of chitosan (5.5–6.5), which restricted its application to the absorption enhancer seriously. For example, the reduction of TEER of Caco-2 cell monolayers was found after the apical incubation with chitosan hydrochloride and chitosan glutamate at a pH of 6.20, but no decrease of TEER, which is a good measurement of the tightness of the junctions between the cells, was observed at a pH of 7.40. At this pH, both chitosan salts (hydrochloride, glutamate) did not form clear solutions. In agreement with the results of the TEER experiments, no increase in the transport of the hydrophilic model compound [^14^C]-mannitol was found at a pH of 7.40 after incubation with these chitosan salts [17]. Therefore, the potential use of chitosans, as absorption enhancer in the more basic environments of intestine, was limited.

Chitosan derivatives have been suggested as promising excipients for absorption enhancement of GI drug in cases in which additional physicochemical properties in the polymer structure were desirable. New types of chitosan molecular or derivatization by introducing small chemical groups to the chitosan structure, for instance, alkyl or carboxymethyl groups could all drastically increase the solubility of chitosan at physiological pH values.

Chito-oligosaccharide (COS), a new type of chitosan molecular, had remarkable solubility in water at physiological pH due to its low molecular weights. Gao et al. (2008) [16] reported the effect of chitosan oligomers on the intestinal absorption of hydrophilic macromolecular drugs, such as insulin and fluorescein isothiocyanate-labeled dextrans (FDs), and found that the bioavailability of insulin as well as FDs could be all increased by about 2.5 times by chitosan hexamer from *in situ* loop method in rats.

Besides, when N-substitution or O-substitution with alkyl groups (i.e., -CH3 groups) for chitosan could increase the aqueous solubility of chitosan without affecting its cationic character, substitution with moieties bearing carboxyl groups can yield polymers with polyampholytic properties. We also found previously that the P_app_ value of Lucifer yellow was improved significantly and TEER was decreased largely compared with the control group as 6-0-CMCs were added in HBSS at a pH of 6.20 or 7.40 from *in vitro* Caco-2 cell (unpublished).

However, no studies have been carried out to examine the effects of COS and CMCs on the intestinal absorption of poorly absorbable compounds of hydrophilic small molecular, such as FTA and CHA.

Therefore, similar to chemical drug development, the current study arms to demonstrate a systemic biopharmaceutics and pharmacokinetics characterization of the two major active ingredients from *Flos Lonicerae* - *Fructus Forsythiae* herb couple for the further development of an improved oral dosage form of *Flos Lonicerae* - *Fructus Forsythiae* herb couple preparations. The specific objectives of the current study include: (1) To improve the intestinal absorption and bioavailability of FTA and CHA by using chitosan derivatives-COS and CMCs based on the *in vitro* Caco-2 cell, *in situ* single-pass intestinal perfusion and *in vivo* pharmacokinetics study characteristics of the two major active ingredients from *Flos Lonicerae* - *Fructus Forsythiae* herb couple preparations; (2) To investigate whether pharmacological effect−antiviral activity of *Flos Lonicerae* - *Fructus Forsythiae* herb couple preparations could be improved by chitosan derivatives.

## Materials and Methods

### Ethics Statement

All procedures had the approval of the Animal Ethics Committee of the Nanjing University of Chinese Medicine.

### Reagents and Chemicals

**Figure 2:**
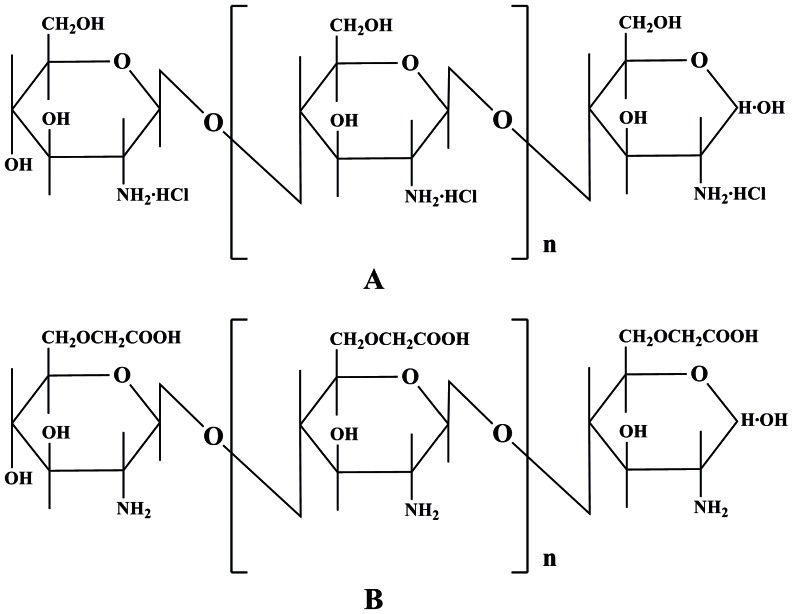
Chemical structure of CMC and COS.

FTA (98% pure) was purchased from Shanghai Nature Standard Co., Ltd. CHA and Scutellarin (using as internal standard, IS) were purchased from National Institute for the Control of Pharmaceutical and Biological Products. COS (average molecular weight 1.5 KDa), HCMC, MCMC and LCMC were all purchased from Qingdao Honghai Bio-tech Co., Ltd., and the chemical structure and physicochemical properties of these chitosan derivatives were listed in Fig. 2 and Table 2. Three *Flos Lonicerae*-*Fructus Forsythiae* preparations (product A-Shuang-Huang-Lian tablet, product B-Yin-Qiao-Jie-Du tablet and product C-Qin-Re-Jie-Du oral liquid manufactured by Harbin third pharmaceutical factory, product D-Fufang Jin-Huang-Lian granule and product E-Fufang Qin-lan oral liquid were purchased from HeiLongJiang ZBD pharmaceutical Co., Ltd.

**Table 2:** Physicochemical properties of various CMCs.

6-OCMCs	Viscosity(1%, 20°C)(mpa⋅s)	Substitutiondegree (%)	pH
HCMC	54	95.5	7.16
MCMC	22	96.5	7.2
LCMC	10	97.1	6.8

Trifluoroacetic acid, Lucifer yellow (LY) and DMSO were purchased from Sigma Chemical Co. (St. Louis, MO). Phosphoric acid, acetic acid, formic acid, methanol and acetonitrile (HPLC grade) were purchased from Merck (Merck, Germany), and water was purified by a Milli-Q water purification system (Millipore, Bedford, MA, USA). All other chemicals and reagents were of analytical grade.

Dulbecco’s modified Eagle’s medium (DMEM), fetal bovine serum (FBS), 0.05% trypsin-EDTA, penicillin-streptomycin and non-essential amino acids were obtained from GibcoBRI, Life and Technologies, USA. Collagen type I, sodium pyruvate, MTT (3-(4,5-dimethylthiazole-2-yl)-2,5-diphenyl tetrazolium bromide) and trypsin_TPCK (Tosylamide Phenylethyl Chloromethyl Keton-treated Trypsin) were purchased from Sigma Chemical Co. (St. Louis, MO, USA). HBSS (Hank’s balanced salt solution) and PBS (Phosphate Buffered Saline) were purchased from Sigma Chemical Co. (St. Louis, MO, USA). Culture cell inserts for 6 well plates (CCI, 137435) were purchase from Nalge Nunc International. (Roskilde, Denmark).

The human colorectal cancer cell lines (Caco-2, HCT116) were bought from cell bank (Chinese Academy of Sciences). Madin-Darby canine kidney cell lines (MDCK cell, KG067) were purchased from Keygen biotech Co., Ltd. The influenza virus strain, A/PR8/34(H1N1)was purchased from Chinese Academy of Preventive Medicine.

Male Sprague-Dawley (SD) rats (∼250 g) were supplied by the Experimental Animal Center of Nanjing University of Chinese Medicine (Certificate No.SCXK2008-0033). The experimental procedures were in compliance with the animal ethics committee of the Nanjing University of Chinese Medicine.

### *In vitro* Caco-2 Monolayer Model

Caco-2 cells were cultured in high glucose DMEM with 10% fetal bovine serum, 1% nonessential amino acids. Cells were cultured in a humidified atmosphere of 5% CO_2_ at 37°C. After reaching 80% confluens, Caco-2 cells were harvested with 0.05% trypsin-EDTA solution and seeded on top of CC inserts in 6-well plates, which has a surface area of 4.2 cm^2^, at a density of 1.0×10^5^ cells/cm^2^. The protocols for cell culture in Transwell inserts were similar to those described previously [2].

Hank’s balanced salt solution (HBSS) was used as the transport buffer for the transport study in Caco-2 cell monolayer model. It was prepared by dissolving 9.5 grams of commercial available HBSS powder in 1000 mL water. The pH value of the buffer was adjusted to pH 6.0 by 85% of phosphoric acid.

MTT test was used to estimate the potential cytotoxicities of the studied FTA, CHA and chitosan derivatives toward Caco-2 cells. The Caco-2 cells were seeded onto a 96-well plate at a seeding density of 5×10^4^ cells/well in DMEM culture medium and cultured at 37°C for 24 h. Subsequently, the culture medium was replaced with 100 µL of FTA, CHA or chitosan derivatives (combined with or without FTA or CHA) dissolved in HBSS (pH 6.0) at different studied concentrations (6.25, 12.5, 25, 50, 100 µM for FTA or CHA; 0.008, 0.016, 0.03125, 0.0625, 0.125, 0.25, 0.5, 1 mg/mL for chitosan derivatives with or without addition of 10 µM of FTA or 60 µM CHA). Blank HBSS (pH 6.0) was employed as a negative control. Then the 96-well plate was incubated at 37°C for 24 h. Thereafter, 20 µL of 5 mg/mL MTT solution in HBSS was added to each well and the plate was incubated for another 4 h. The solutions in each well were then removed followed by dissolving the remained formazan crystals in the cells with 200 µL of DMSO. The absorbance of the mixture in the 96-well plate was then measured with a Kinetic microplate reader (Molecular Devices) at 570 nm. The cytotoxicity of each compound was calculated as the percentage of the absorbance relative to that of the negative control.

Cell culture experiments were described previously [2]. Briefly, after culture medium was aspirated, the cell monolayers were washed three times with blank HBSS. The transepithelial electrical resistance (TEER) values of cell monolayers were measured, which were more than 250 Ω* m^2^. The monolayers were incubated with the blank HBSS for 1 h with 37°C. Thereafter the incubation medium was aspirated. Afterwards, a solution containing the compound was loaded onto the apical side. The amounts of transported compound were measured as a function of time by UPLC-MS method described previously [11]. Donor samples (400 µL) (Apical side) and receiver samples (400 µL) (Basolateral side) were taken at different times (typically 1 h), followed by the addition of 400 µL drug donor solution to the donor side (AP) and 400 µL of blank buffer to the receiver side (BL). The samples were taken at 0, 1, 2, 3 and 4 h after incubation. At the end of the transport experiment, integrity of the monolayer was monitored by TEER value.

### Rat *in situ* Single Pass Intestinal Perfusion Study

The perfusion buffer was isotonic and composed of 118 mM of NaCl, 4.7 mM of KCl, 1.2 mM of MgSO_4_⋅H_2_O, 2.5 mM of CaCl_2_, 1.2 mM of KH_2_PO_4_ and 25 mM of NaHCO_3_. All the above chemicals were dissolved in water followed by adjusting the pH to 6.0 by concentrated phosphate acid. Phenol red at 20 µg/ml was added to the perfusate as a non-absorbable marker.

Rat *in situ* single pass intestinal perfusion model was set at previously described by us [11], [18]. Briefly, male SD rats (body weight: 250–300 g) were fasted overnight with free access to water. The rats were anesthetized with 20% urethane solution (6 mg/Kg). A midline abdominal incision was made and the small intestine was exposed. The bile duct was ligated in order to avoid bile secretion into the perfusate. For the regional absorption of drugs, three intestinal sections were isolated and cannulated (all were 10 cm long): duodenum, jejunum and ileum. Each segment was rinsed with normal saline at 37°C for 20 min until the washing appeared clear. After that, the perfusion solution of drugs as solvent was connected to the each segment and perfusing through each part of the three intestine sections. At the beginning of 30 minutes, the circulation rate was 0.2 ml/min controlled by a peristaltic pump to pre-balance, then, perfusate samples were collected.

Solutions containing 10 µM FTA and 60 µM CHA were perfused through the intestinal lumen to investigate the permeabilities of the two studied compounds in the rats *in situ* intestinal perfusion model. In addition, 10 µM FTA and 60 µM CHA with the addition of chitosan derivatives were also perfused through the intestinal lumen to investigate the effect of chitosan derivatives on the permeabilities of the two studied compounds. The perfusate samples were collected at 30–60, 60–90, 90–120 and 120–150 min, and stored at −80°C refrigerator until analysis.

### Rat *in vivo* Pharmacokinetics Study

FTA and CHA was freshly prepared in freshly normal saline solution at a concentration of 10 mg·mL^−1^ of FTA, 60 mg·mL^−1^ of CHA for intravenous administration and 1 mg·mL^−1^ of FTA, 6 mg·mL^−1^ of CHA for oral administration. Product A, B, C, D, E extracts were also dissolved in saline to give the concentration of 50% (*v*/*v*) immediately prior to drug administration. The contents of FTA and CHA were determined to be 2 mg·mL^−1^ and 12 mg·mL^−1^ of extracts, respectively, using an HPLC assay.

Male SD rats (∼250 g) were obtained from Experimental Animal Center of Nanjing University of Chinese Medicine and kept in an environmentally controlled breeding room (temperature: 20±2°C, relative humidity: 60±5%) for 1 week. The animals were fasted for 12 h prior to drug administration.

The rats were randomly divided into 38 groups shown in Table 3 with five rats in each group to receive various administrations at a single oral dose (10 mL·kg^−1^) by gastric gavage or intravenous dose (1 mL·kg^−1^) by rapid injection via the catheter. After dosing for 0, 5, 10, 15, 20, 30, 40, 55, 70, 100, 160, 250, 480 min, blood was collected from the pre-intubated catheter and put into tubes with heparin sodium injection (10 µL) and ascorbic acid (2 µg) at predetermined time points. Subsequently, plasma was prepared by centrifugation at 1816×*g* for 7 min and stored at −80°C for further analysis.

**Table 3:** The dosage regimen of FTA and CHA in rat.

Drug	Chitosan derivative	Dosage of chitosan derivatives(mg·kg^−1^)	Administration route
FTA	–	–	Intravenously
FTA	–	–	Orally
CHA	–	–	Intravenously
CHA	–	–	Orally
Product A extract	–	–	Orally
Product B extract	–	–	Orally
Product C extract	–	–	Orally
Product D extract	–	–	Orally
Product E extract	–	–	Orally
FTA	COS	12.5	Orally
FTA	COS	25	Orally
FTA	COS	50	Orally
CHA	COS	12.5	Orally
CHA	COS	25	Orally
CHA	COS	50	Orally
FTA	LCMC	12.5	Orally
FTA	LCMC	25	Orally
FTA	LCMC	50	Orally
CHA	LCMC	12.5	Orally
CHA	LCMC	25	Orally
CHA	LCMC	50	Orally
FTA	MCMC	12.5	Orally
FTA	MCMC	25	Orally
FTA	MCMC	50	Orally
CHA	MCMC	12.5	Orally
CHA	MCMC	25	Orally
CHA	MCMC	50	Orally
FTA	HCMC	25	Orally
FTA	HCMC	50	Orally
FTA	HCMC	100	Orally
CHA	HCMC	25	Orally
CHA	HCMC	50	Orally
CHA	HCMC	100	Orally
Product A extract	COS	25	Orally
Product B extract	COS	25	Orally
Product C extract	COS	25	Orally
Product D extract	COS	25	Orally
Product E extract	COS	25	Orally

### Effect of Chitosan Derivatives on the GI Membrane Toxicity Evaluated by Morphological Observation

After *in vivo* pharmacokinetics experiments were carried out for 8 h, the GI was wash with HBSS (pH 7.4) and the segments were removed and immersed in the 4% neutral paraform aldehyde buffer and fixed. Vertical sections were prepared, stained with hematoxylin-eosin, and examined by light microscopy. To be able to describe possible changes in the structure of the tissue, tissues were studied and evaluated for morphological changes using the following parameters:

Measurement of the distance between the nucleus and the apical membrane in enterocytes with a measuring-rod. Five measurements in each villus and five villi were measured in each slide. The villi were randomly chosen and measurements were all conducted at the tips of the villi.

Villus index was used for duodenum, jejunum and ileum and crypt index for colon. Measurement of height (villi) or depth (crypt) and width (one-half of height or depth) was done with a measuring-rod of five villi/crypts in each slide that were randomly chosen. Height/depth was divided with width to receive the index.

### Effect of *Flos Lonicerae* - *Fructus Forsythiae* Herb Couple Preparations with or without COS on Influenza Virus

MDCK cells were grown in DMEM as described previously [18], supplemented with 10% FBS and 1% Pen/Strep at 37°C in a humidified incubator. The media was changed two to three times per week. The influenza virus was propagated in MDCK cells in the presence of 1 µg/mL of Trypsin_TPCK to create the working stock. During antiviral evaluations, media supplemented with FBS was sucked out and the cell washed with PBS and then it was treated as needed. The media supplemented with Trypsin_TPCK was added.

Serum after administration orally into *Flos Lonicerae* - *Fructus Forsythiae* herb couple preparations with or without COS at the dosages of 25 mg/kg was added to the MDCK cells after the propagation with influenza virus. The cells were incubated at 37°C for 48 h before viability testing by measuring the conversion of MTT as described in section **3.1.3.**

### Calculation

For Caco-2 monolayer model, the apparent permeability coefficient (*P*_app_) was calculated as *P*_app_ = [(dQ/dt)]/[A×C], d*Q*/d*t* (µg/S) was the flux rate, *A* was the effective surface area of the cell monolayer (4.2 cm^2^), and *C*_0_ (µg/mL) was the initial drug concentration in the donor chamber. For rat single-pass intestinal perfusion *in situ* model, the concentration of perfusion fluid was calculated as *C*_out(corrected)_ = *C*_out_ *PR*_in_/*PR*_out_ and the effective permeability coefficient (*P*_eff_) was calculated as *P*_eff_ = *Q*ln[*C*_in_/*C*_out(corrected)_]/2πrL. *C*_out(corrected)_ was effluent drug concentration with correction; *C*_out_ was effluent drug concentration without correction; *C_in_* was influent drug concentration; *PR*_in_ was influent phenol red concentration; *PR*_out_ were effluent phenol red concentration; *Q* was perfusate flow rate; *r* was radius of intestinal segment and *l* was intestinal segment length. Inhibition rate =  [*OD*(drug)−*OD*(model)]/[*OD*(control)−*OD*(model)].

### Pharmacokinetic Analysis

The peak concentrations (*C*_max_) and the time to reach the peak concentrations (*T*_max_) were determined directly from the plasma concentration–time profiles. The area under the curve (*AUC*) was calculated by the trapezoidal method from time zero to the final sampling. The absorption enhancement ratios of drugs with or without enhancers were calculated as *Absorption enhancement ratio* = *AUC*_with enhancer_/*AUC*_ control (without enhancer)._

### Statistical Analysis

Statistical significance in the *P*_app_, *P*_eff_ values, pharmacokinetic parameters and inhibition rate index obtained from various treatment groups was estimated by the analysis of variance (Student t-test) or one-way ANOVA. A *p* value of less than 0.05 was considered to be significantly different. All data were expressed as mean±SD.

## Results

### Effect of Different Chitosan Derivatives on the *P*_app_-value of FTA and CHA in the Apical-to-basolateral (AP-BL) Direction from *in vitro* Caco-2 monolayer Model

Caco-2 cells were exposed to various concentrations of absorption enhancers (0.008, 0.016, 0.03125, 0.0625, 0.125, 0.25, 0.5, 1 mg/mL) with addition of 10 µM FTA and 60 µM CHA for 24 h. It was shown that HCMC, MCMC, LCMC and COS at different concentrations were all safe for the Caco-2 cells.

**Figure 3:**
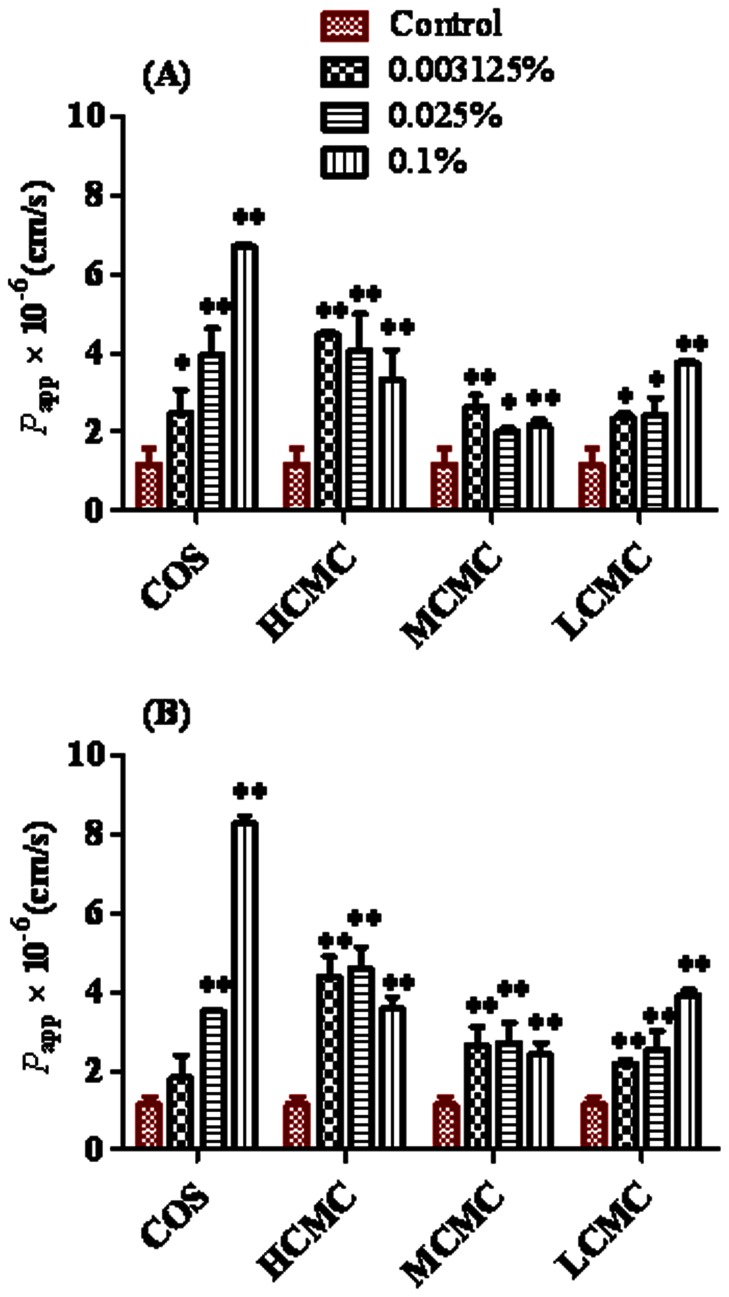
Effects of CMCs and COS on absorption parameters of FTA and CHA in Caco-2 cell ***in vitro*** **model.** Results are expressed as the mean ± S.D. (^*^) *P*<0.05 and (^**^) *P*<0.01 compared with the control group (A: FTA; B: CHA).

As shown in Fig. 3, COS and LCMC at the same low, medium and high concentrations caused a significant, concentration-dependent increase in the *P*_app_-value for FTA and CHA compared to the control group (*p*<0.05). The highest *P*_app_-value for FTA and CHA was increased to 573% (6.71±0.06)×10^−6 ^cm/s and 722% (8.28±0.19) ×10^−6 ^cm/s with addition of 0.1% (w/v) of COS, and increased to 321% (3.76±0.06)×10^−6 ^cm/s and 342% (3.92±0.13) in the presence of 0.1% (w/v) of LCMC. However, the addition of HCMC and MCMC induced the greatest increase in the *P*_app_-value at a relatively low concentration (0.003125%) for FTA and at a medium concentration (0.025%) for CHA. The highest *P*_app_-value for FTA was increased to 381% (4.47±0.08)×10^−6 ^cm/s and 225% (2.64±0.30)×10^−6 ^cm/s with addition of 0.003125% (w/v) of HCMC and MCMC, and The highest *P*_app_-value for CHA was increased to 401% (4.60±0.54)×10^−6 ^cm/s and 222% (2.54±0.47)×10^−6 ^cm/s in the presence of 0.025% (w/v) of HCMC and MCMC. The results indicated that the intestinal absorption of FTA and CHA can be improved by chitosan derivatives. Meanwhile, the absorption enhancing effect of HMCM and MMCM might be almost saturable up to 0.003125% (*w*/*v*) for FTA and 0.025% (*w*/*v*) for CHA.

### Effect of Chitosan Derivatives on the Absorption of FTA and CHA in Rat from *in situ* Intestinal Perfusion Model

**Figure 4:**
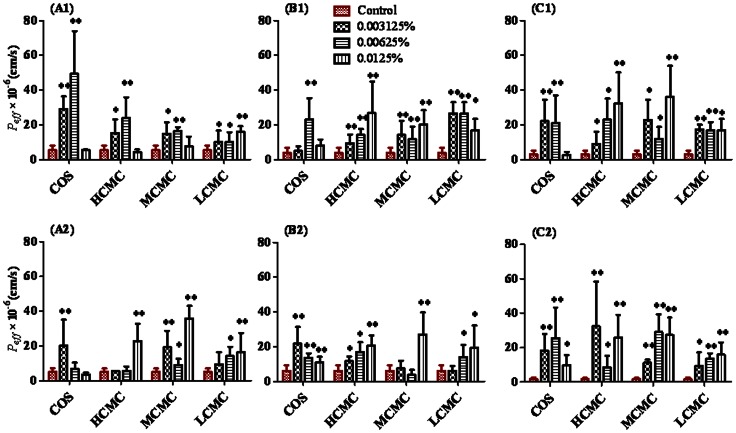
Effects of CMCs and COS on absorption parameters of FTA and CHA in rat single pass intestinal perfusion via duodenum (A), jejunum (B) and ileum (C) ***in situ*** **model.** Results are expressed as the mean ± S.D. (^*^) *P*<0.05 and (^**^) *P*<0.01 compared with the control group (A1, B1, C1: FTA; A2, B2, C2: CHA).

As shown in Fig. 4, COS at moderate dose could enable FTA to reach the maximum *P*_eff_-values in duodenum, jejunum and ileum, but the maximum *P*_eff_-values of CHA in the three intestine sites could be reached when COS was at low dose; HCMC resulted in dose-dependent absorption in jejunum and ileum, but in duodenum HCMC at moderate dose produced maximum *P*_eff_-values of FTA, and HCMC led to dose-dependent *P*_eff_-values of CHA in duodenum and jejunum, but in ileum HCMC at low dose yielded maximum absorption; MCMC at low dose could enable FTA to reach the maximum *P*_eff_-values in duodenum, jejunum and ileum, but the maximum *P*_eff_-values of CHA in the three intestine sites could be reached when MCMC was at high dose; Besides, LCMC gave *P*_eff_-values of FTA in a dose-dependent manner in duodenum and ileum, but at low dose made FTA be absorbed maximally in jejunum. However, LCMC led to dose-dependent absorption of FTA in the three intestine sites. The results indicated that the intestinal absorption of FTA and CHA can be enhanced by chitosan derivatives. Meanwhile, the absorption enhancing effect of chitosan derivatives for FTA and CHA might be saturable in different intestine sites.

### Effect of Chitosan Derivatives on the Oral Bioavailabilities of FTA and CHA from *in vivo* Pharmacokinetics Study

**Figure 5:**
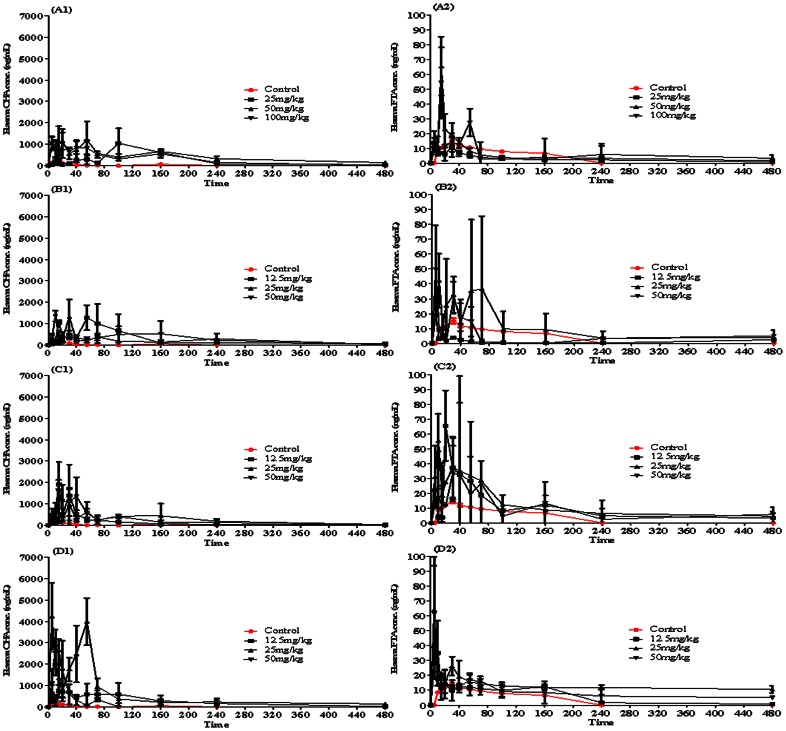
Plasma concentration-time profiles of FTA (A2, B2, C2 and D2) and CHA (A1, B1, C1 and D1) with CMCs and COS after administration to the rat gastrointestine by *in vivo* pharmacokinetics study. Results are expressed as the mean±S.D. of 3–5 experiments (A1, A2: HCMC; B1, B2: MCMC; C1, C2: LCMC; D1, D2: COS).

As shown in Fig. 5 and Table 4, In the CMC groups, 50 mg/kg HCMC, 25 mg/kg MCMC and 25 mg/kg LCMC displayed the largest *AUC* values of CHA in three groups; On the other hand, 25 mg/kg MCMC and 25 mg/kg LCMC showed the largest *AUC* values of FTA in three groups. However, the absorption-enhancing ability of CHA and FTA in COS at the dosage of 25 mg/kg group was higher significantly than that of other groups. Therefore, these findings indicated that 25 mg/kg COS would be the promising enhancer for improving the bioavailability of FTA and CHA in rats.

**Table 4:** Effects of chitosan derivatives on the absorption of FTA and CHA by *in vivo* pharmacokinetics study.

		FTA		CHA	
		AUC0-480 min(ng⋅min/mL)	Ratio	AUC0-480 min(ng⋅min/mL)	Ratio
Control		1710.6±98.0	−	8629.7±3859.0	−
HCMC	100 mg/kg	2033.0±526.1*	1.4	142299.7±20523.6**	16.5
	50 mg/kg	2918.0±1108.1*	1.7	206727.5±25568.3**	24.0
	25 mg/kg	1307.0±267.0	0.8	127791.7±28400.0**	14.8
MCMC	50 mg/kg	2203.0±660.4	1.3	135409.0±56350.0**	15.7
	25 mg/kg	4485.0±2489.0**	2.6	140506.0±43126.3**	16.3
	12.5 mg/kg	3232.2±461.2	1.9	92111.0±31224.0**	10.7
LCMC	50 mg/kg	4592.7±2257.2*	2.7	116372.0±47007.7**	13.5
	25 mg/kg	5744.7±3362.2*	3.2	123045.0±44796.6**	14.3
	12.5 mg/kg	4629.0±3805.8*	2.7	71708.0±7957.78**	8.3
COS	50 mg/kg	3143.0±572.1**	1.8	152641.5±72581.0**	17.7
	25 mg/kg	6286.0±1329.7**	3.7	296980.0±36456.0**	34.4
	12.5 mg/kg	2874.7±564.9**	1.7	52938.0±3520.0**	6.1

### Effect of COS at the Dosage of 25 mg/kg on the Intestinal Membrane Toxicity by Morphological Observation of GI Tissues

**Figure 6:**
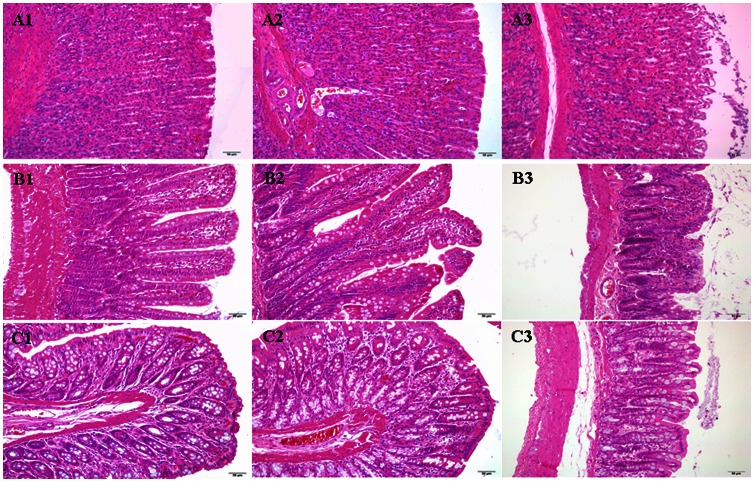
HE photomicrographs of rat gastric and intestinal tissue sections after oral administration of COS at the dosage of 25 mg/kg. All panels represent cross-sections of gastric and intestinal tissues. Jejunum represents small intestine. The original magnification was 20× object lens for stomach and jejunum, 40× object lens for colon. A, B and C represent stomach, jejunum and colon, respectively. A1–C1 (PBS); A2–C2 (COS) and A3–C3 (Triton X-100).

Morphological observation of the intestinal mucosa after administration orally COS at the dosage of 25 mg/kg was shown in Fig. 6 (A1–A3: stomach, B1–B3: jejunum, C1–C3: colon). The results clearly indicated that COS at the dosage of 25 mg/kg did not cause any significant change in the morphology of the GI membrane, although we found that 200 mg/kg Triton X-100 as a positive control could cause mucosal damage seriously. Besides, in these micrographs, all morphological parameters described below can be viewed.

**Figure 7:**
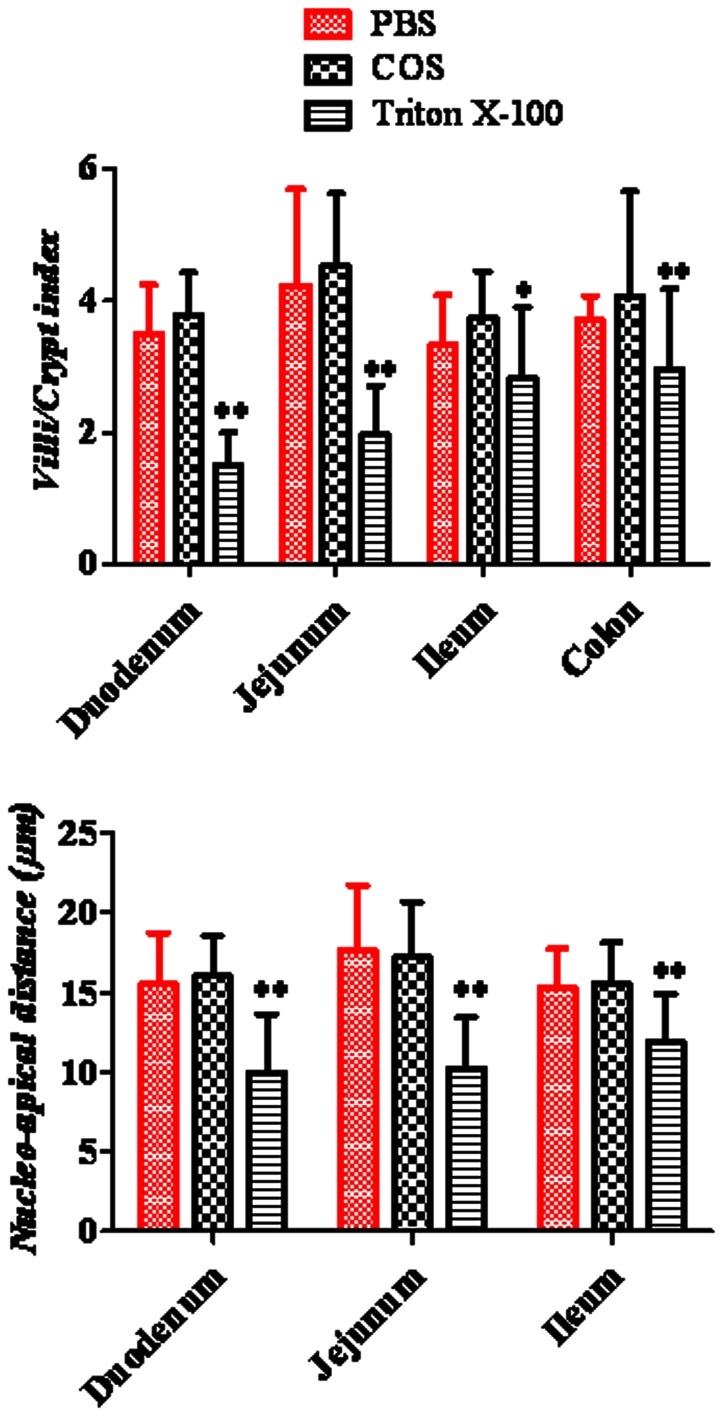
Nucleo-apical distance (µm) for duodenum, jejunum and ileum and villi index for duodenum, jejunum and ileum, and crypt index for colon for COS at the dosage of 25 **mg/kg.** Results are presented as mean values ±S.D. (*n* = 4–6). (^*^) *P*<0.05 and (^**^) *P*<0.01 compared with the PBS group.

Nucleo-apical distance was measured in duodenum, jejunum and ileum (Fig. 7). Nucleo-apical distance in the presence of 25 mg/kg COS was not significantly decreased in duodenum, jejunum and ileum, although there was a remarkable decrease in nucleo-apical distance after administrating orally 200 mg/kg Triton X-100 as a positive control.

Villi index calculated for the small intestinal segments and crypt index calculated for colon were presented also in Fig. 7. The result indicated that villi/crypt index in the presence of 25 mg/kg COS was not obviously decreased in duodenum, jejunum, ileum and colon, although there was a remarkable decrease in nucleo-apical distance after administrating orally 200 mg/kg Triton X-100 as a positive control.

### Effect of COS at the Dosage of 25 mg/kg on the Bioavailability of *Flos Lonicerae* - *Fructus Forsythiae* Herb Couple Preparations

**Figure 8:**
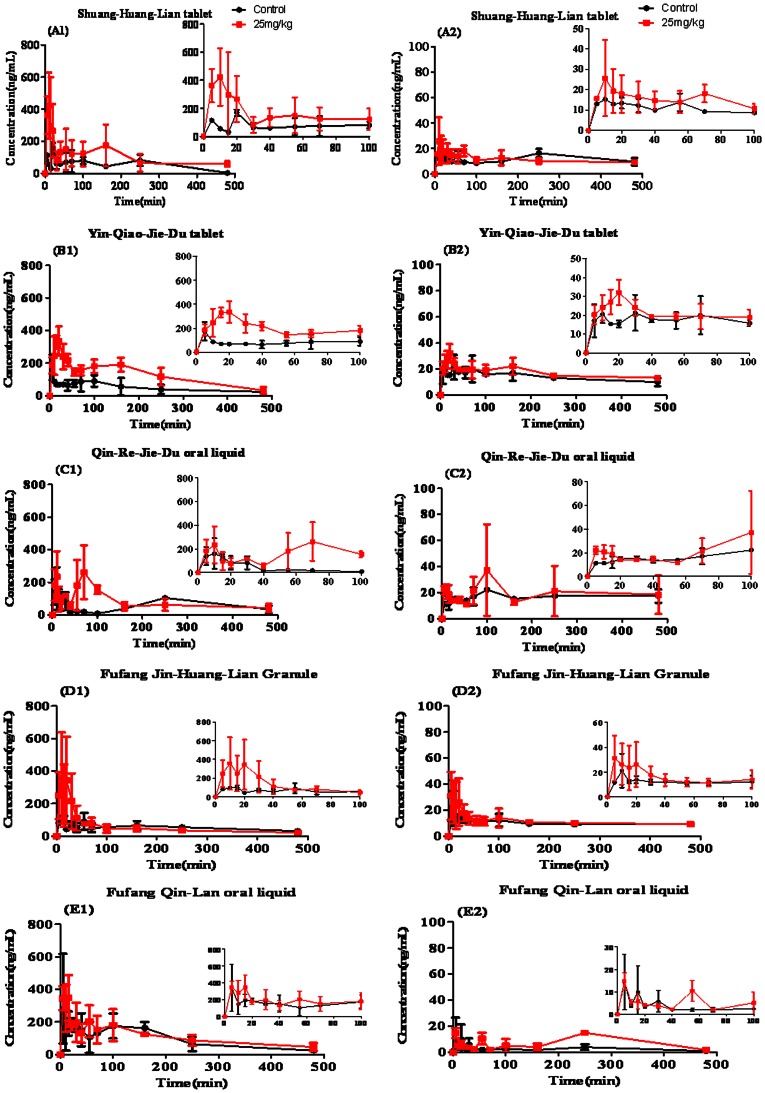
Plasma concentration-time profiles of FTA (A2, B2, C2, D2 and E2) and CHA (A1, B1, C1, D1 and E1) in *Flos Lonicerae - Fructus Forsythiae* herb couple preparations with COS at the dosage of 25 mg/kg after administration to the rat gastrointestine by *in vivo* pharmacokinetics study. Results are expressed as the mean±S.D. of 3–5 experiments.

As shown in Fig. 8 and Table 5, it was seen that the absorption-enhancing ratio of FTA and CHA were 1.85 and 2.7 in Shuang-Huang-Lian tablet, 1.22 and 2.6 in Yin-Qiao-Jie-Du tablet, 2.38 and 1.6 in Fufang Jin-Huang-Lian, 1.67 and 1.4 in Qin-Re-Jie-Du oral liquid, 1.96 and 1.2 in Fufang Qin-Lan oral liquid, respectively. It was indicated that the oral bioavailability of main active ingredients in *Flos Lonicerae - Fructus Forsythiae* herb couple preparations could be improved significantly by COS at the suitable dosage compared with that of control.

**Table 5:** Effects of COS at the dosage of 25 mg/kg on the oral bioavailability of FTA and CHA in *Flos Lonicerae* - *Fructus Forsythiae* herb couple preparations.

	FTA			CHA		
	AUC(control)	AUC(absorptionenhancer)	Ratio	AUC(control)	AUC(absorption enhancer)	Ratio
Shuang-Huang-Lian tablet	3563.0±668.0	6604.3±614.3*	1.85	27020.0±4518.0	72996.0±31582.2*	2.7
Yin-Qiao-Jie-Du tablet	6731.3±650.5	8195.3±591.1*	1.22	23910.0±12524.3	62083.5±10313.2**	2.6
Fufang Jin-Huang-Lian Granule	2619.3±399.2	6222.0±5206.0*	2.38	25501.3±3404.0	32302.0±11585.2*	1.6
Qin-Re-Jie-Du oral liquid	6694.0±1781.6	11203.75±8458.3*	1.67	28468.0±4452.8	39608.0±7849.3**	1.4
Fufang Qin-Lan oral liquid	1014.1±373.5	1988.1±1180.5*	1.96	45672.0±212.1	53129.0±2891.4	1.2

### Effect of *Flos Lonicerae* - *Fructus Forsythiae* Herb Couple Preparations with or without COS on Influenza Virus

**Figure 9:**
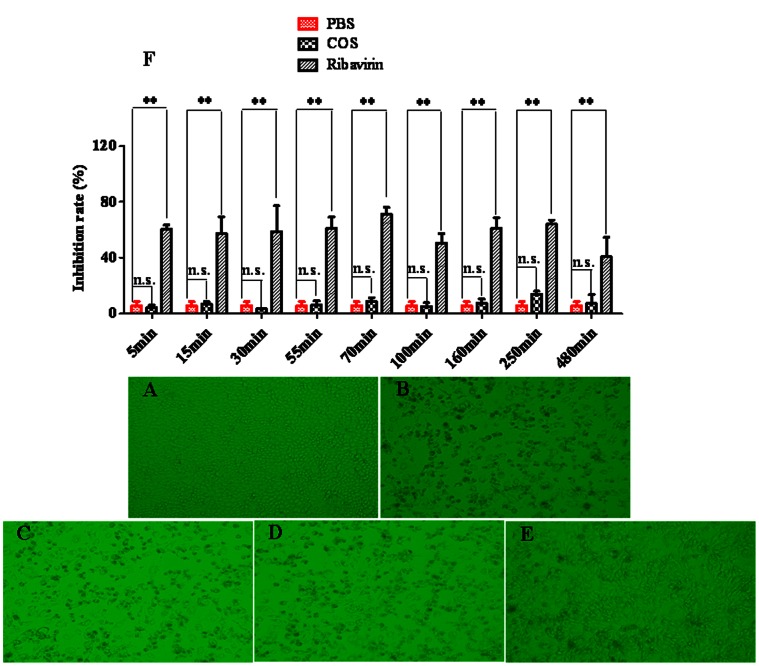
Cytopathogenic effect observed on influenza virus infections in MDCK cells. (A: Normal group; B: Virus group; C: PBS group; D: COS group; E: Ribavirin as positive control group; F: Inhibition rate of COS and ribavirin on influenza virus); Inhibition rate was assayed with MTT 48 hours later and expressed as percentage of controls (data ±S.D. *n* = 8). (^*^) *P*<0.05, (^**^) *P*<0.01 and (N.S.) no significant difference.

**Figure 10:**
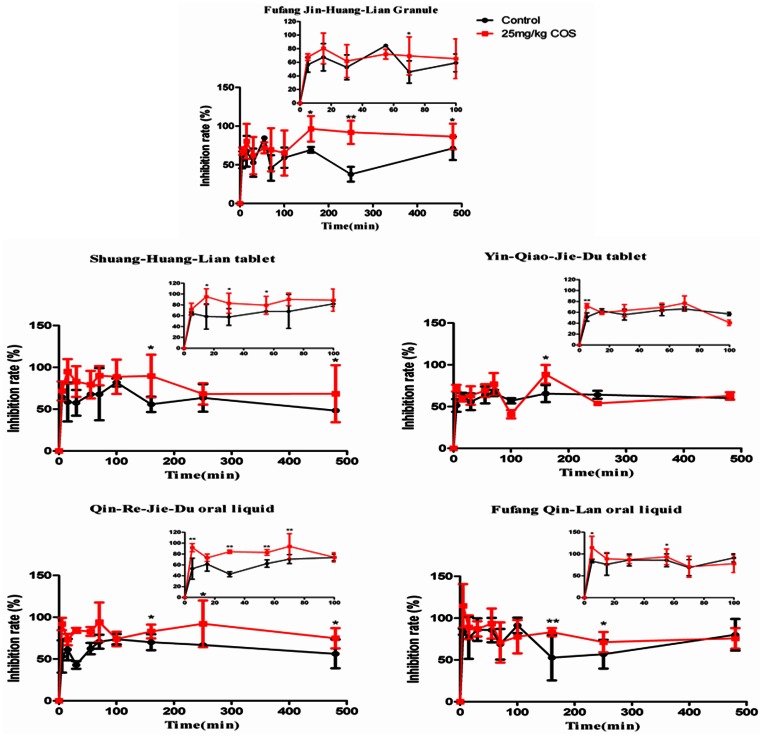
Effect of *Flos Lonicerae - Fructus Forsythiae* herb couple preparations with or without COS at the dosage of 25 mg/kg on influenza virus. Inhibition rate was assayed with MTT 48 hours later and expressed as percentage of controls (data ±S.D. *n* = 8). (^*^) *P*<0.05 and (^**^) *P*<0.01.

As shown in Fig. 9, the antiviral model was built successfully, and the inhibition rate of COS group was no significant compared with that of PBS group, although there was a remarkable increase in inhibition rate value after administrating orally 20 mg/kg ribavirin as a positive control. However, the difference of antiviral activity between *Flos Lonicerae* - *Fructus Forsythiae* herb couple preparations with or without COS was significant from Fig. 10. The inhibition rate of *Flos Lonicerae* - *Fructus Forsythiae* herb couple preparations with COS at dosage of 25 mg/kg was higher than that of without COS, which indicated that the pharmacological effects such as antiviral effect of *Flos Lonicerae* - *Fructus Forsythiae* herb couple preparations could be significantly improved by addition of COS.

## Discussion

*Flos Lonicerae* - *Fructus Forsythiae* herb couple is the basic components of Chinese herbal preparations such as Shuang-Huang-Lian tablet, Yin-Qiao-Jie-Du tablet, Fufang Jin-Huang-Lian Granule, Qin-Re-Jie-Du oral liquid and Fufang Qin-Lan oral liquid *etc*., shown in Table 1, which is extensively used for treating acute upper respiratory tract infection caused by virus or bacterial infection in clinical practice. And oral preparations of *Flos Lonicerae* - *Fructus Forsythiae* herb couple is usually more accepted for patients than injections owning to their elimination of pain and discomfort, and lower costs to produce oral formulations, but their clinical effect is unsatisfactory compared with that of injections, which becomes one of the most limited points in the development of Chinese medicine preparations. Whether the low bioavailability of oral *Flos Lonicerae* - *Fructus Forsythiae* herb couple preparations resulted in the poor efficacy or whether efficacy could be improved as the absorption of active ingredients in *Flos Lonicerae* - *Fructus Forsythiae* herb couple preparations was enhanced has been investigated in this study. FTA and CHA were two main ingredients in *Flos Lonicerae* - *Fructus Forsythiae* herb couple preparations. In previous reports, Zhang et al. (2002) [20] and Shang et al. (2011) [1] conducted physicochemical studies of FTA and CHA, respectively, showing that they were highly hydrophilic compounds almost completely dissociated in biological fluids. This physicochemical property of the drugs has been elucidated by us demonstrating that the low permeability in the intestinal mucosa was an important reason for its reported low bioavailability, and paracellular route was crossed mainly [2], [10]. Then CMCs and COS, chitosan derivatives possessing non-toxic biocompatible polymeric as TJs enhancers obtained from chitin, were used to improve the intestinal absorption of FTA and CHA in *Flos Lonicerae* - *Fructus Forsythiae* herb couple preparations shown in Fig. 8 [21]. Meanwhile, as shown in Fig. 9, the influenza virus was propagated in MDCK cells. And we found that the MDCK cells in Fig. 9 (B) as virus group have been infected by influenza virus, compared with cells in Fig. 9 (A) as normal group. Besides, MDCK cells in Fig. 9 (D) as positive group were infected less than that in virus group, which all indicated that the antiviral model was constructed successfully. The inhibition rate of COS group was not significant compared with that of PBS group, although there was a remarkable increase in inhibition rate after administrating orally 20 mg/kg ribavirin as a positive control shown in Fig. 9 (F). However, as shown in Fig. 10, the inhibition rate for influenza virus of *Flos Lonicerae* - *Fructus Forsythiae* herb couple preparations with COS at the dosage of 25 mg/kg was higher significantly than that of the preparations without COS. All evidence above could support the hypothesis that caffeic acid derivatives like FTA and CHA could be important markers for controlling the pharmacology of *Flos Lonicerae* - *Fructus Forsythiae* herb couple preparations. Nevertheless, further investigation on other ingredients related to their pharmacological effects should be examined for controlling the quality of preparations better.

We also found that the absorption enhancing effect of chitosan derivatives for improving the intestinal absorption of FTA and CHA was affected by their concentrations (Figs. 3 and 4). Besides, a maximal absorption enhancing effect of HCMC, MCMC, LCMC and COS to CHA and FTA was observed at dosages of 50, 25, 25, and 25 mg/kg, respectively (Table 4 and Fig. 5), not at higher doses. This was consistent with Fig. 4 showing that the *P*_eff_-values of FTA and CHA were enhanced by COS and CMCs, but exhibiting saturable effect in single pass intestinal perfusion model *in situ*. Gao et al. (2008) [16] reported the absorption enhancing effect of chitosan hexamer for FD4 was dependent on its concentration, but its absorption enhancing effect was almost saturable up to 0.5% (*w*/*v*) from *in situ* loop method. Artursson et al. (1994) [22] also reported that the enhancing effect of chitosan on the absorption of mannitol was almost saturable up to 0.5% (*W/V*) in Caco-2 cells *in vitro*. Therefore, our present results were consistent with the previous reports. Chitosan and its derivatives have some optimal concentrations to show the greatest absorption enhancing effects for improving the intestinal absorption of poorly absorbable drugs in rats.

It was shown in Table 4 and Fig. 5 that CMCs and COS not only enhanced the intestinal absorption of CHA but also improved the intestinal absorption of FTA in rats, although the bioavailability of CHA and FTA were improved highest by COS at the dosage of 25 mg/kg, not CMCs. Possible reasons for absorption-enhancing ability of CHA and FTA improved more by COS than CMCs could be that 1) chitosan had antimicrobial activity [23], [24]; 2) CHA was mainly metabolized by intestinal bacteria after administration orally reported by Konishi et al. (2004) [10], and 3) the intestinal bacteria metabolism of CHA and FTA was inhibited higher by COS than CMCs via intestinal bacterial incubation *in vitro*, although the intestinal metabolism by bacteria of FTA was lower than that of CHA (unpublished). This was consistent with the previous report [16] showing that the improved intestinal absorption of hydrophilic small molecular by chitosan was due to inhibition of degradation of hydrophilic small molecular, thereby increasing the intestinal absorption from the intestine. Besides, Illum et al. (1994) [25] proposed that the mechanism of absorption enhancement was mucoadhesion through ionic interactions with negatively charged groups of glycocalix. It was shown in Table 4 that the enhancing ability of COS at the dosage of 25 mg/kg was higher than that of CMCs, although CMCs showed more viscosity than COS. At last, from the Caco-2 cells, it was found that the TEER value moderately decreased in the presence of chitosan derivatives compared with control (unshown), indicating that chitosan derivatives might loosen the tight junction, thereby increasing the permeability of drugs via paracellular pathway, which was consistent with the data reported by Schipper at al. (1997) [26] showing that the binding and absorption enhancement of chitosan on epithelial cells were mediated through their positive charges, and that the interactions of chitosans with the cell membrane resulted in a structural reorganization of tight junction-associated proteins followed by enhanced transport via the paracellular pathway. And it was seen from Fig. 3 that the enhancing ability of COS at the concentration of 0.1% (*W/V*) for CHA and FTA was higher than that of other CMCs at the same concentration in Caco-2 cell model *in vitro*.

When absorption enhancers are applied in clinical use, their potential local toxicity should be considered. We observed in Fig.7 that there was no significant change of GI mucosa, and the morphological parameters, nucleo-apical distance and villi index [27], [28] in all small intestinal segments and crypt in colon shown in Fig. 8 also changed little in the presence of COS compared with PBS group. This indicated that COS was a promising candidate for the enhancement of absorption of drugs using GI delivery system due to its non-toxicity and unidirectional immediate enhancement action [29], biocompatibility, and biodegradability characteristics [30]. Besides, we found that the contents of active ingredients such as FTA and CHA *in vitro* in *Flos Lonicerae* - *Fructus Forsythiae* herb couple preparations with COS at the dosage of 25 mg/kg were not degraded compared with control from LC/MS system (unpublished), which may prove the compatibility of COS and TCM.

### Conclusion

Current findings from *in vitro*, *in situ* and *in vivo* consistently demonstrated that COS at the dosage of 25 mg/kg improved the intestinal absorption and bioavailabilities of FTA and CHA to the greatest extent, and was safe for gastrointestine from morphological observation. Addition of COS at the dosage of 25 mg/kg in the *Flos Lonicerae* - *Fructus Forsythiae* herb couple products further proved the usefulness of COS for enhancing the oral absorption of FTA and CHA in *Flos Lonicerae* - *Fructus Forsythiae* herb couple products. Besides, treatment with *Flos Lonicerae* - *Fructus Forsythiae* herb couple products with COS at dosage of 25 mg/kg prevented MDCK cell damage after propagation with influenza virus better than that of control.

All findings above not only identify the role of COS as the absorption enhancer for the improved oral delivery of *Flos Lonicerae* - *Fructus Forsythiae* herb couple, but also demonstrate the importance of biopharmaceutics and pharmacokinetics characterization of the active ingredients in the further dosage form development of traditional Chinese medicine products.

## References

[1] Shang XF, Pan H, Li MX, Miao XL, Ding H (2011) *Lonicera japonica Thunb*.: Ethnopharmacology, phytochemistry and pharmacology of an important traditional Chinese medicine. J Ethnopharmacol 138: 1–21. 21864666 10.1016/j.jep.2011.08.016 PMC7127058

[2] Zhou W, Qin KM, Shan JJ, Ju WZ, Liu SJ, et al (2012) Improvement of intestinal absorption of forsythoside A in weeping forsythia extract by various absorption enhancers based on tight junctions. Phytomedicine 20: 47–58. 23089157 10.1016/j.phymed.2012.09.014

[3] Ma YY, Zhang ZW, Li HW, Sun JH, Xu CY (2010) Effects of forsythoside A on the Expression of IFN-α and Mx1. Sci Agr Sin 43: 3237–3243.

[4] Qu H, Zhang Y, Wang Y, Li B, Sun W (2008) Antioxidant and antibacterial activity of two compounds (forsythiaside and forsythin) isolated from Forsythia suspense. J Pharm Pharmacol 60: 261–267. 18237475 10.1211/jpp.60.2.0016

[5] Kuang HX, Xia YG, Yang BY, Liang J, Zhang QB, et al (2009) A new caffeoyl phenylethanoid glycoside from the unripe fruits of forsythia suspense. Zhong Guo Tian Ran Yao Wu 7: 278.

[6] Hu KJ, Sun KX, Wang JL (2001) Inhibited effect of chlorogenic acid on virus in vitro. J Harrbing Med Univ 35: 430–432.

[7] Zou Y, Liao S, Shen W, Liu F, Tang C, et al (2012) Phenolics and antioxidant activity of mulberry leaves depend on cultivar and harvest month in southern China. Int J Mol Sci 13: 16544–16553. 23443117 10.3390/ijms131216544 PMC3546706

[8] Wang GN, Pan RL, Liao YH, Chen Y, Tang JT, et al (2010) An LC-MS/MS method for determination of forsythiaside in rat plasma and application to a pharmacokinetic study. J Chromatogr B 878: 102–106. 10.1016/j.jchromb.2009.11.029 19945919

[9] Ye JX, Wei W, Quan LH, Liu CY, Chang Q, et al (2010) An LC–MS/MS method for the simultaneous determination of chlorogenic acid, forsythiaside A and baicalin in rat plasma and its application to pharmacokinetic study of Shuang-huang-lian in rats. J Pharm Biomed Anal 52: 625–630. 20153596 10.1016/j.jpba.2010.01.035

[10] Konishi Y, Kobayashi S (2004) Transepithelial transport of chlorogenic acid, caffeic acid and their colonic metabolites in intestinal Caco-2 cell monolayers. J Agric Food Chem 52: 2518–2526. 15113150 10.1021/jf035407c

[11] Zhou W, Di LQ, Wang J, Shan JJ, Liu SJ, et al (2012) Intestinal absorption of forsythoside A in *in situ* single-pass intestinal perfusion and *in vitro* Caco-2 cell models. Acta Pharmacol Sin 33: 1069–1079. 22773077 10.1038/aps.2012.58 PMC4011322

[12] Salama NN, Eddington ND, Fasano A (2006) Tight junction modulation and its relationship to drug delivery. Adv Drug Deliv Rev 58: 15–28. 16517003 10.1016/j.addr.2006.01.003

[13] Uchiyama T, Sugiyama T, Quan YS, Kotani A, Okada N, et al (1999) Enhanced permeability of insulin across the rat intestinal membrane by various absorption enhancers: their intestinal mucosal toxicity and absorption-enhancing mechanism of n-lauryl-β-D-maltopyranoside. J Pharm Pharmacol 51: 1241–1250. 10632081 10.1211/0022357991776976

[14] Fetih G, Habib F, Okada N, Fujita T, Attia M, et al (2005) Nitric oxide donors can enhance the intestinal transport and absorption of insulin and [Asu(1,7)]-eel calcitonin in rats. J Control Release 106: 287–297. 15982776 10.1016/j.jconrel.2005.05.017

[15] Yamamoto A, Uchiyama T, Nishikawa R, Fujita T, Muranishi S (1996) Effectiveness and toxicity screening of various absorption enhancers in the rat small intestine: effects of absorption enhancers on the intestinal absorption of phenol red and the release of protein and phospholipids from the intestinal membrane. J Pharm Pharmacol 48: 1285–1289. 9004192 10.1111/j.2042-7158.1996.tb03937.x

[16] Gao Y, He L, Katsumi H, Sakane T, Fuiita T, et al (2008) Improvement of intestinal absorption of insulin and water-soluble macromolecular compounds by chitosan oligomers in rats. Int J Pharm 359: 70–78. 18450395 10.1016/j.ijpharm.2008.03.016

[17] Jonker C, Hamman JH, Kotze AF (2002) Intestinal paracelluar permeation enhancement with quaternised chitosan: in situ and in vitro evaluation. Int J pharm 238: 205–213. 11996824 10.1016/s0378-5173(02)00068-6

[18] Du Q, Di LQ, Shan JJ, Liu TS, Zhang XZ (2009) Intestinal absorption of daphnetin by rats single pass perfusion in situ. Yao Xue Xue Bao 44: 922–926. 20055163

[19] Mehrbod P, Motamed N, Tabatabaian M, Soleimani ER, Amini E (2009) *In vitro* antiviral effect of “Nanosilver” on influenza virus. Drug 17: 88–93.

[20] Zhang LW (2002) Extraction method and biological activities of forsythiaside. Taiyuan: Shanxi University.

[21] Hejazi R, Amiji M (2003) Chitosan-based GI delivery systems. J Control Release 89: 151–165. 12711440 10.1016/s0168-3659(03)00126-3

[22] Artursson P, Lindmark T, Davis SS, Illum L (1994) Effect of chitosan on the permeability of monolayers of intestinal epithelial cells (Caco-2). Pharm Res 11: 358–1361. 10.1023/a:1018967116988 7816770

[23] Liu BY, Cao SY, Li BL, Yang ZY (2003) Study on antimicrobial activity of chitoologosaccharide. Chin J Biochem Pharm 24: 73–75.

[24] Yuan W, Yin XQ, Feng YH, He YM, Pan MD, et al (2007) Research progress of preparation and antimicrobial activity of carboxymethyl chitosan. Food Res Development 28: 185–189.

[25] Illum L, Farraj NF, Davis SS (1994) Chitosan as a novel nasal delivery system for peptide drugs. Pharm Res 11: 1186–1189. 7971722 10.1023/a:1018901302450

[26] Schipper NG, Olsson S, Hoogstraate JA, Varum KM, Artursson P (1997) Chitosan as absorption enhancers for poorly absorbable drugs 2: mechanism of absorption enhancement. Pharm Res 14: 923–929. 9244151 10.1023/a:1012160102740

[27] Thomson AB, Keelan M, Clandinin MT, Walker K (1986) Dietary fat selectively alters transport properties of rat jejunum. J Clin Invest 77: 279–288. 3944255 10.1172/JCI112288 PMC423337

[28] Rafter JJ, Eng VW, Furrer R, Medline A, Bruoe WR (1986) Effects of calcium and pH on the mucosal damage produced by deoxycholic acid in the rat colon. Gut 27: 1320–1329. 3792915 10.1136/gut.27.11.1320 PMC1434073

[29] Bernkop-Schnürch A, Dünnhaupt S (2012) Chitosan-based drug delivery system. Eur J Pharm Biopharm 81: 463–469. 22561955 10.1016/j.ejpb.2012.04.007

[30] Barry BW (1983) Percutaneous absorption. In: Barry, B.W. (ed.). Dermatological Preparations, Marcel Dekker, New York, 127–233.

